# Investigation of the levels of circulating miR-29a, miR-122, sestrin 2 and inflammatory markers in obese children with/without type 2 diabetes: a case control study

**DOI:** 10.1186/s12902-021-00829-z

**Published:** 2021-08-03

**Authors:** Khalid M. Mohany, Osamah Al Rugaie, Osama Al-Wutayd, Abdullah Al-Nafeesah

**Affiliations:** 1 Department of Medical Biochemistry, College of Medicine, Assiut University, P.O. Box, Assiut, 71515 Egypt; 2 Department of Basic Medical Sciences, Unaizah College of Medicine and Medical Sciences, Qassim University, Unaizah, Saudi Arabia; 3 Department of Family and Community Medicine, Unaizah College of Medicine and Medical Sciences, Qassim University, Unaizah, Saudi Arabia; 4 Department of Pediatrics, Unaizah College of Medicine and Medical Sciences, Qassim University, Unaizah, Saudi Arabia

**Keywords:** MiR-29a, miR-122, Sestrin 2, Childhood obesity, T2DM

## Abstract

**Aim:**

The present work investigated serum levels of miR-29a, miR-122 and sestrin2 in obese children with/without type-2-diabetes mellitus (T2DM), and their correlations with inflammatory, metabolic and anthropometric parameters.

**Methods:**

The study included 298 children, divided into: G1 (control, *n* = 136), G2 (obese without diabetes, *n* = 90) and G3 (obese with T2DM, *n* = 72). Metabolic and anthropometric parameters, miR-29a, miR-122 relative expressions, and sestrin2, high sensitivity C-reactive protein (hsCRP), interleukin-6 (IL-6), and tumor necrosis factor-α (TNF-α) levels were measured by their specific methods. The data was processed and analyzed by SPSS V.26 using the corresponding tests. After testing the variables’ normality, Kruskal–Wallis one-way-ANOVA, Spearman correlations coefficient were used.

**Results:**

Significant higher serum miR-29a, miR-122, IL-6, hsCRP and TNF-α and lower sestrin2 levels were found in G2 and G3 than G1 and in G3 than G2 (p= > 0.001 for all). Especially in G3, miR-29a and miR-122 levels correlated positively while sestrin2 levels correlated negatively with waist circumference and BMI percentiles, serum levels of LDL-cholesterol, triacylglycerol, total cholesterol, HbA1c%, glucose, insulin, c-peptide, homeostatic model assessment-insulin resistance (HOMA-IR), IL-6, hsCRP and TNF-α.

**Conclusion:**

The change in the serum miR-29a, miR-122 and sestrin2 levels in obese children with/without T2DM may suggest a possible role of these biomarkers in the pathogenesis of childhood obesity and their accompanied complications e.g. inflammations and T2DM. Also, further studies are required to test drugs that antagonize the action miR-29a and miR-122 or upregulate sestrin2 in the management of these cases.

**Supplementary Information:**

The online version contains supplementary material available at 10.1186/s12902-021-00829-z.

## Introduction

Globally, the prevalence of childhood and adolescence obesity has increased markedly in the last decade [1, 2]. About 30% of Saudi Arabia children were reported to be obese or overweight [3, 4]. Obese children have a high risk of developing many cardiac and metabolic complications such as type 2 diabetes mellitus (T2DM) either during their childhood or, later during their adulthood [5, 6].

Indeed, both obesity and T2DM are considered as systemic inflammatory conditions [7, 8]. Obesity is associated with dysregulation in the adipocyte-derived cytokines that leads to a generalized low inflammatory condition and insulin resistance [9, 10]. In this regard, many previous studies were conducted to understand the pathogenesis of the obesity accompanied inflammations in order to prevent, control and manage them [7–10].

MicroRNAs (miRs) are short non-coding sequences of nucleotides that can modulate the process of adipogenesis and many of its related consequences [11]. Abnormal expressions of microRNAs have been reported by many previous studies in obese individuals with/without diabetes [5, 12]. Of these microRNAs, miR-29a was found to correlate with insulin resistance and inflammatory parameters in obese adults with/without T2DM [13–15]. Also, miR-122 has been considered a good marker for obesity and its related complications. It correlated positively with insulin resistance in subjects without diabetes [16, 17].

Sestrin2 is an inducible protein that guards the cell from stress injury and keeps up optimum cell functions, metabolism, and endurance [18]. A decline in the cellular level of sestrin2 is associated with oxidative damage, mitochondrial dysfunction, impaired glucose tolerance and diabetes mellitus [18]. The investigations of sestrin2 levels in patients with metabolic syndrome (e.g. obesity with/without T2DM) have revealed controversial results [18–20].

As many of the previous studies have tested the levels of miR-29a, miR-122 and sestrin 2 mainly in adults and some of their results were controversial, the present work aimed to measure these levels in obese children with/without T2DM and compared them to those of healthy non-obese controls. In addition, it investigated the correlation of these levels with various inflammatory, metabolic and anthropometric parameters.

### Subjects and methods

About 1415 consecutive children who visited the primary health care units in Unaizah governorate, Qassim area, Saudi Arabia between January and August 2019 approached for participation in the present study. A number of 1117 of them were excluded according to the exclusion criteria mentioned later. The eligible children were 298; 195 males (65.5%) and 103 females (34.5%), their ages were 9 to 15 years. All the study participants’ parents were acquainted with the study aim and gave a written informed consent. Medical histories were collected and examinations were done for all children. The children’s waist circumference (WC) and body mass index (BMI) percentiles were calculated and the child was considered obese when his/her WC ≥ 90th percentile and BMI ≥95 percentile for age and sex [3]. The participants were divided into three groups; G1 (non-obese healthy control group, *n* = 136), G2 (obese without diabetes group, *n* = 90) and G3 (obese with T2DM, *n* = 72). Diagnostic criteria of cases with T2DM included a random plasma blood glucose ≥200 mg/dl, or fasting blood glucose of ≥126 mg/dl, or oral glucose tolerance test with blood sugar ≥200 mg/dl hours post ingestion, or glycosylated hemoglobin percentage (HbA1c%) > 6.5% [21].

The G3 participants had been managed only by life-style adjustments (those who had received any drug interventions were excluded from the study). Also, exclusion criteria included those who presented with T1DM, genetic or endocrinal disorders, inflammatory, or any other systemic illness [12].

### Sampling and laboratory analysis

Five ml of fasting blood samples were withdrawn from all children. One ml was used to measure HbA1c% in the whole blood by glycated hemoglobin kit (Sigma–Aldrich, St. Louis, MO, USA). The remaining portion was left to be clotted then centrifuged for 10 min at 3000 rpm and the sera were collected in aliquots and stored at − 80 °C till the assay. Fasting serum glucose levels were measured by glucose oxidase activity assay kit (Colorimetric) (abcam Cat# ab219924). Colorimetric methods were used to estimate serum total cholesterol (Spectrum Diagnostic Egypt, Cat# 230003), HDL-C (Spectrum Diagnostic Egypt, Cat# 266002), serum triacylglycerol (Spectrum Diagnostic Egypt, Cat# 314003). LDL-C was estimated by Friedewald formula [22]. Insulin levels were measured by electrochemiluminescence assay and C-peptide was measured by human C-Peptide quantikine enzyme-linked immunosorbent assay (ELISA Kit DICP00, R&D systems). Homeostasis model assessment of insulin resistance (HOMA-IR) was calculated after Matthew et al. [23].

The levels of sestrin2 in the serum were assessed by the sestrin 2 ELISA method (MYBIOSOURCE, San Diego, CA 92195–3308, USA, # MBS2024978) with detection range between 0.15 ng/ml - 10 ng/ml. Human interleukin-6 (IL-6) Solid Phase Sandwich ELISAKit (R&D systems) was used to measure serum IL-6 levels with assay range b 0.2–10 pg/ml. A latex-enhanced immunoturbidimetric assay (Diazyme Laboratories, 12,889 Gregg Ct, Poway, CA 92064, USA, Cataloge# DZ135A-K) was used to measure the high-sensitivity C-reactive protein (hsCRP) with detection range 0.20–20 mg/l. The TNFα levels were measured by ELISA consistent with the accompanied protocol (Quantikine ELISA Kit R&D Systems, Minneapolis, MN, USA).

### RNA extraction

Circulating miRNAs were isolated and purified using miRNeasy kit (ThermoFisher scientific, Ambion® PureLink® miRNA Isolation Kit, USA, Cat# K157001). Briefly, the sera were centrifuged in a spin cartridge for 1 min at 12000 rpm. Ethanol (96–100%) was added and mixed to the flow through to a final concentration of 70%. Then, 500 μL sample was transferred to a second spin cartridge and centrifuged again for 1 min at 12000 rpm. The miRNAs were bound to spine cartilage while the flow through was discarded. The spin cartridge was then washed with 500 μL wash buffer mixed with ethanol (prepared by adding 40 ml 96–100% ethanol to 10 ml wash buffer) then centrifuged for 1 min at 12000 rpm (this step was repeated once). The spin cartridge was then centrifuged at the maximum speed for 1 min to eliminate any residual wash buffer. The miRNAs were eluted with 50–100 μL RNase-free water then incubated at room temperature for 1 min. The purified miRNAs were stored at − 80 °C till being used [24].

### Complementary DNA (cDNA)

Reverse transcription was done cDNA RT Kit (Applied Biosystems, Foster City, CA, USA, Cat. No: 438814) according to the manufacturers’. The miScript HiSpec Buffer was used. The samples were diluted with RNase-free water to even out the total amount of RNA. Twenty μL reverse transcription reaction, 8 μL of the master mix, 12 μL of RNA template and RNase-free were mixed in 0.2 ml PCR tubes and incubated at 37 °C for sixty minutes then at 95 °C for five minutes in thermal cycler (Applied Biosystems).

### Real time polymerase chain reaction (qRT-PCR)

It was done using TaqMan® MiR RT Kit (Applied Biosystems, Foster City, CA, USA). Two and half μL universal master mix, 0.25 μL primer and probe set, 0.33 μL cDNA, and 1.92 μL H_2_O were mixed to make a 5 μL reaction volume. qRT-PCR was done at 50 °C for 2 min and 95 °C for 10 min, followed by 40 cycles at 95 °C for 15 s and 60 °C for 1 min. The cel-miR-39 was used as an internal control during the quantification using specific stem-loop primers for miR-29a and miR-122 [5, 12].

The analysis of the results was performed using Sequence Detection Software version 2.3 (Applied Biosystems). The difference in the expression levels of miR-29a and miR-122 between samples was calculated using the 2^-ΔΔCT^ method [5, 12].

### Statistical analysis

The collected data was analyzed by SPSS (v26). The Shapiro-Wilk test was used to evaluate the normality of continuous variables. Kruskal-Wallis one-way-ANOVA was used to compare them among the three studied groups. The correlations between different studied continuous variables levels were tested by Spearman’s correlations coefficient. A 2-tailed *p* < 0.05 was considered statistically significant.

## Results

### Comparison of different variables in the 3 studied groups

The current study found significant high levels of miR-29a, miR-122, IL-6, hsCRP and TNF-α in obese (G2) and obese with T2DM (G3) groups compared to the control (G1) group (Table 1) and in obese with T2DM (G3) group compared to obese (G2) group. On the other hand, the levels of sestrin 2 were significantly low in obese (G2) and obese with T2DM (G3) groups compared to the control (G1) group and in obese with T2DM (G3) group compared to obese (G2) group (Table 1).

**Table 1:** Comparison of different variables among G1, G2 and G3

		Healthy control group (G1; n = 136)	Obese group (G2; n = 90)	Obese with type 2 diabetes group (G3; ***n*** = 72)
**Gender** **n (%)**	**Male**	88 (64.7%)	62 (68.9%)	45 (62.5%) ^**a, ¶**^
	**Female**	48 (35.3%)	28 (31.1%)	27 (37.5%) ^**a, ¶**^
**Age (years)**		12.1 ± 1.3	12.2 ± 1.4	12.2 ± 1.2 ^**¶**^
**WC percentile**		72.3 ± 7.2	95.9 ± 2.9	96.1 ± 2.9 ^*^
**BMI percentile**		79.1 ± 6.8	96.8 ± 1.5	96.7 ± 1.4 ^*^
**HDL-c (mg/dl)**		41.2 ± 2.9	35.7 ± 8.5	34.0 ± 9.5 ^*^
**LDL-c (mg/dl)**		120.4 ± 21.4	126.1 ± 25.6	132.4 ± 17.0
**TAG (mg/dl)**		107.8 ± 19.6	112.7 ± 21.3	142.4 ± 37.1
**Cholesterol (mg/dl)**		183.2 ± 21.6	184.4 ± 26.6	194.9 ± 21.1
**Glucose (mg/dl)**		81.7 ± 12.7	96.8 ± 20.6	106.5 ± 25.7 ^**#**^
**HbA1c (%)**		4.4 ± 0.7	6.0 ± 1.8	7.9 ± 1.9 ^**#**^
**Insulin (μg/ml)**		7.6 ± 1.7	10.2 ± 0.7	10.6 ± 1.2^*^
**C-Peptide (ng/ml)**		2.8 ± 0.4	3.0 ± 0.3	3.1 ± 0.3 ^**♣**^
**HOMA-IR**		1.5 ± 0.4	2.4 ± 0.5	2.8 ± 0.6 ^**#**^
**miR-29a R**		1.5 ± 0.3	1.9 ± 0.2	2.3 ± 0.3 ^**#**^
**miR-122 R**		1.6 ± 0.3	1.9 ± 0.6	2.6 ± 0.9 ^**#**^
**Sestrin2 (ng/mL)**		5.8 ± 1.8	4.1 ± 2.6	2.9 ± 1.4 ^**#**^
**hsCRP (mg/dl)**		0.8 ± 0.1	1.3 ± 0.1	1.4 ± 0.2 ^**#**^
**IL-6 (pg/ml)**		1.8 ± 0.4	2.2 ± 0.8	2.7 ± 1.1 ^**#**^
**TNF-α (pg/ml)**		1.1 ± 0.2	1.3 ± 0.1	1.6 ± 0.6 ^**#**^

Unless otherwise indicated, the data is presented as mean ± Standard deviation

WC: waist circumference, BMI: body mass index, HDL-c: high density lipoprotein cholesterol, LDL-c: low density lipoprotein cholesterol, TAG: triacylglycerol, HbA1c: glycosylated hemoglobin, HOMA-IR: Homeostatic model Assessment-Insulin resistance, miR-29a R: microRNA-29a relative expression, miR-122 R: microRNA-122 relative expression, hsCRP: high-sensitivity C-reactive protein, IL-6: interleukin 6, TNF-α: tumor necrosis factor alpha

^a^Chi-square

^**¶**^ the p-values of G1 Vs. G2 Vs. G3, G1 Vs. G2, G1 Vs. G3 and G2 Vs. G3 were non-significant

* the *p*-values of G1 Vs. G2 Vs. G3, G1 Vs. G2, and G1 Vs. G3 were > 0.001 while G2 Vs. G3 were non-significant

# the p-values of G1 Vs. G2 Vs. G3, G1 Vs. G2, G1 Vs. G3 and G2 Vs. G3 were > 0.001

^**♣**^ the p-values of G1 Vs. G2 Vs. G3, G1 Vs. G2, G1 Vs. G3 and G2 Vs. G3 were (0.01, 0.02, 0.009 and 0.44, respectively)

The p-values of G1 Vs. G2 Vs. G3 for LDL-c (0.06), TAG (> 0.001) and Cholesterol (non-significant

### Correlations analysis

#### In the whole sample

**Fig. 1:**
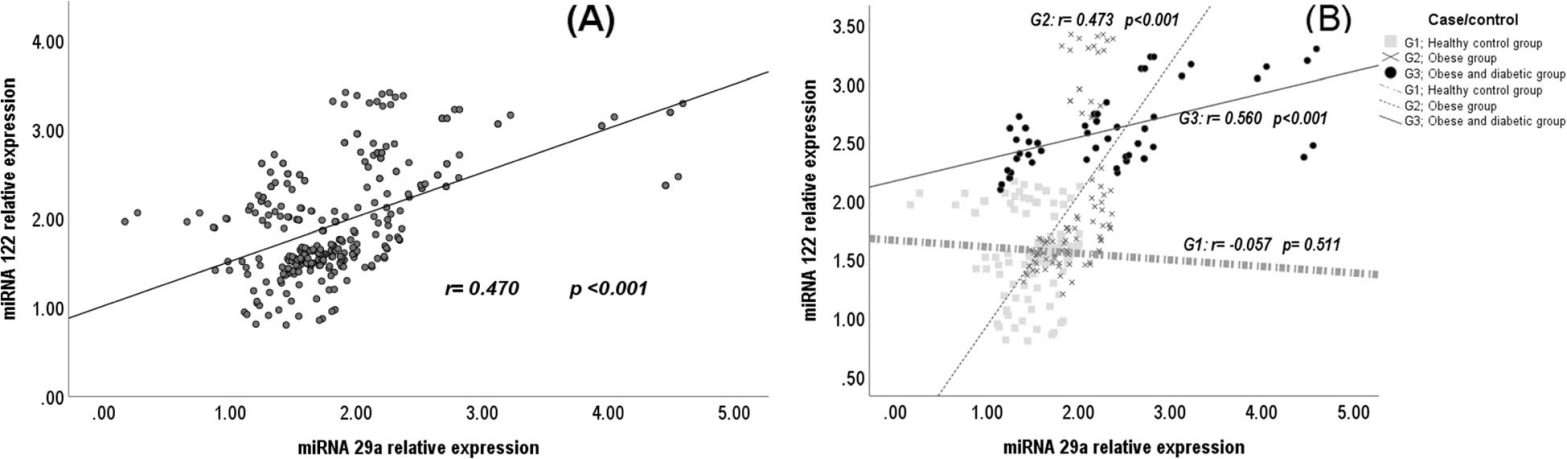
Correlations of microRNA-29a relative expressions and microRNA-122 relative expressions: **(A)** in the whole sample, and **(B)** in G1, G2 and G3

**Fig. 2:**
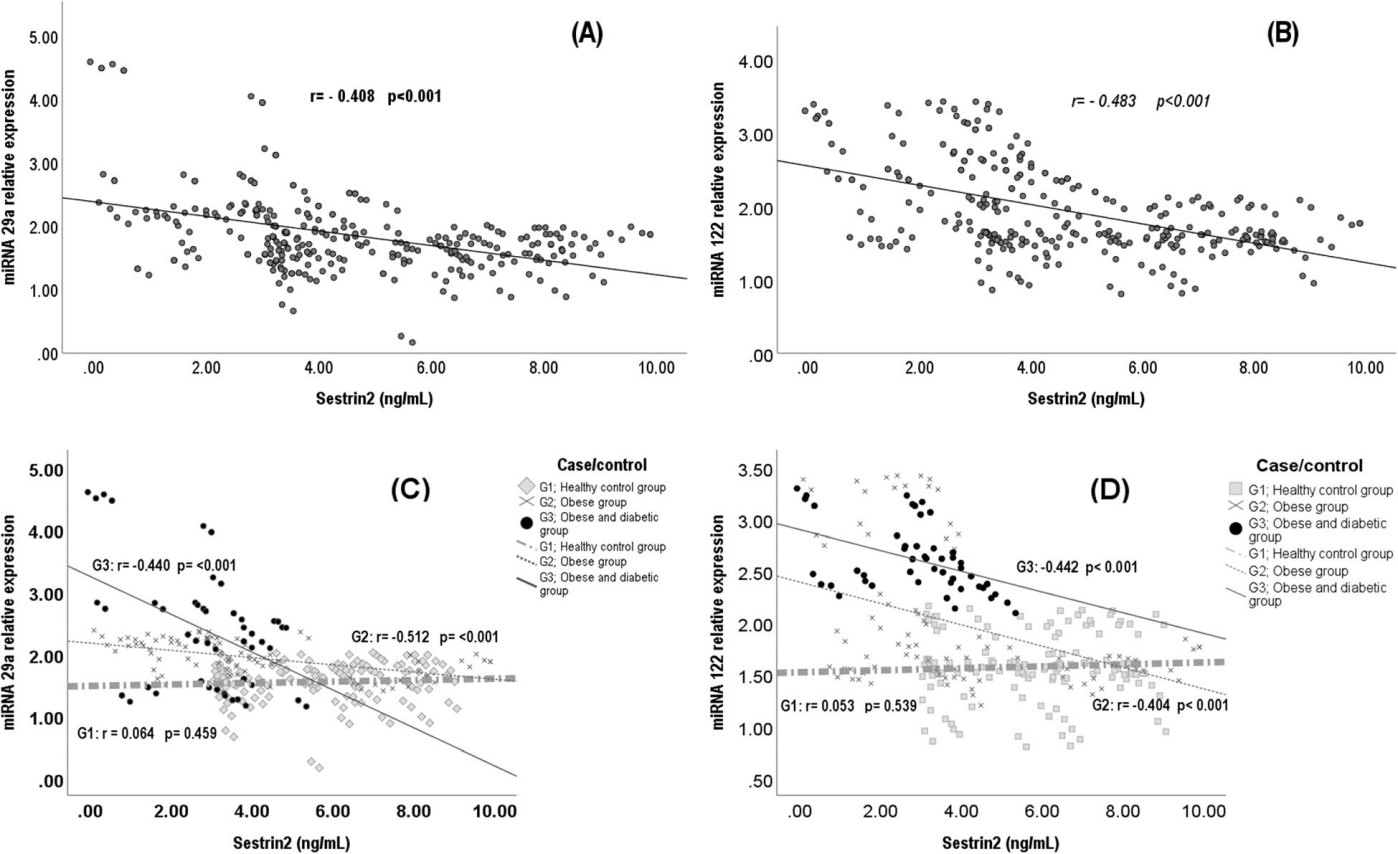
Correlations between sestrin 2 levels and **(A)** miR-29a relative expressions in the whole sample, **(B)** miR-122 relative expressions in the whole sample, **(C)** miR-122 relative expressions in G1, G2 and G3, and **(D)** miR-29a relative expressions in G1, G2 and G3

The serum levels of miR-29a, miR-122, hsCRP, IL-6 and TNF-α correlated positively, while serum sestrin2 levels correlated negatively with WC percentiles and BMI percentiles, and serum levels of LDL-cholesterol, triacylglycerols, HbA1c%, glucose, insulin, c-peptide and with HOMA-IR. Also, the serum miR-29a and miR-122 levels correlated positively with each other (Fig. 1A) and with serum hsCRP, IL-6 and TNF-α levels while correlated negatively with serum sestrin2 levels (Table 2, Fig. 2A and B). On the other hand, serum sestrin2 levels correlated negatively with serum levels of hsCRP, IL-6 and TNF-α (Table 2).

**Table 2:** Correlations of the relative expressions of microRNA-29a and microRNA-122, serum sestrin 2, hsCRP, IL-6 and TNF-α levels with the studied anthropometric and metabolic parameters in whole study sample

	WC percentile	BMI percentile	HDL-c (mg/dl)	LDL-c (mg/dl)	TAG (mg/dl)	Cholesterol (mg/ml)	HbA1c (%)	Glucose (mg/dl)	Insulin (μg/ml)	C-Peptide (ng/ml)	HOMA-IR	hsCRP (mg/dl)	IL-6 (pg/ml)	TNF-α (pg/ml)
**miR-29aR**	0.478^**^	0.458^**^	−0.315^**^	0.460^**^	0.517^**^	0.365^**^	0.570^**^	0.654^**^	0.356^**^	0.254^**^	0.651^**^	0.497^**^	0.267^**^	0.355^**^
**miR-122R**	0.610^**^	0.619^**^	−0.457^**^	0.277^**^	0.384^**^	0.201^♣^	0.468^**^	0.412^**^	0.513^**^	0.293^**^	0.631^**^	0.502^**^	0.427^**^	0.453^**^
**Sestrin2** **(ng/ml)**	−0.495^**^	−0.844^**^	0.308^*^	−0.215^*^	−0.273^**^	− 0.077^♣^	−0.428^**^	− 0.321^**^	−0.331^**^	− 0.227^**^	−0.558^**^	− 0.577^**^	−0.504^**^	− 0.541^**^
**hsCRP (mg/dl)**	0.874^**^	0.815^**^	−0.455^**^	0.231^**^	0.402^**^	0.277^**^	0.672^**^	0.578^**^	0.342^**^	0.366^**^	0.605^**^	1	0.521^**^	0.664^**^
**IL-6 (pg/ml)**	0.440^**^	0.411^**^	−0.364^**^	0.271^**^	0.362^**^	0.271^**^	0.456^**^	0.481^**^	0.358^**^	0.319^**^	0.748^**^		1	0.626^**^
**TNF-α (pg/ml)**	0.529^**^	0.510^**^	−0.456^**^	0.287^**^	0.508^**^	0.289^**^	0.613^**^	0.506^**^	0.469^**^	0.428^**^	0.679^**^			1

The values presented in the table are for the correlation coefficient

^♣^ non-significant correlation

^*^correlation is significant at 0.05

^**^correlation is significant at 0.01

WC: waist circumference, BMI: body mass index, HDL-c: high density lipoprotein cholesterol, LDL-c: low density lipoprotein cholesterol, TAG: triacylglycerol, HbA1c: glycosylated hemoglobin, HOMA-IR: Homeostatic model Assessment-Insulin resistance, miR-29a R: microRNA-29a relative expression, miR-122 R: microRNA-122 relative expression, hsCRP: high-sensitivity C-reactive protein, IL-6: interleukin 6, TNF-α: tumor necrosis factor alpha

#### In control group (G1)

The serum levels of miR-29a correlated positively with serum levels of LDL-cholesterol, triacylglycerols, cholesterol, HbA1c%, glucose, and insulin (Table 3). Its correlations with the serum levels of miR-122 and sestrin2 were non-significant (Figs. 1B and 2C). The serum levels of miR-122 correlated positively with WC percentiles and BMI percentiles and with serum insulin levels while negatively correlated with HDL-cholesterol levels (Table 3). Its correlation with serum sestrin2 levels was non-significant (Fig. 2D). Serum sestrin2 levels correlated positively with HDL-cholesterol. The serum levels of hsCRP correlated positively with BMI percentile and with serum levels of IL-6 and TNF-α while negatively correlated with the serum levels HDL-cholesterol. The serum levels of IL-6 correlated positively with BMI percentile and with the serum levels of TNF-α (Table 3).

**Table 3:** Correlations of the relative expressions of microRNA-29a and microRNA-122, serum sestrin 2, hsCRP, IL-6 and TNF-α levels with the studied anthropometric and metabolic parameters in G1, G2 and G3

			WC percentile	BMI percentile	HDL-c (mg/dl)	LDL-c (mg/dl)	TAG (mg/dl)	Cholesterol (mg/ml)	HbA1c (%)	Glucose (mg/dl)	Insulin (μg/ml)	C-Peptide (ng/ml)	HOMA-IR	hsCRP (mg/dl)	IL-6 (pg/ml)	TNF-α (pg/ml)
**G1; Healthy control group** **(*n*** **= 68)**	**miR-29aR**	***r***	0.163^♣^	0.068^♣^	−0.002^♣^	0.204^*^	0.249^**^	0.238**	0.252**	0.287**	0.206*	−0.132^♣^	0.002^♣^	0.024^♣^	0.051^♣^	0.035^♣^
	**miR-122R**	***r***	0.428^**^	0.515^**^	−0.241^**^	0.105^♣^	−0.138^♣^	0.045^♣^	0.060^♣^	−0.023^♣^	0.207^*^	0.165^♣^	0.145^♣^	0.102^♣^	0.056^♣^	0.069^♣^
	**Sestrin2** **(ng/ml)**	***r***	0.054^♣^	0.055^♣^	0.186^*^	0.125^♣^	−0.079^♣^	0.082^♣^	0.017^♣^	−0.074^♣^	0.089^♣^	−0.014^♣^	0.039^♣^	−0.019^♣^	0.046^♣^	0.014^♣^
	**hsCRP (mg/dl)**	***r***	0.143^♣^	0.207^*^	−0.256^**^	−0.061^♣^	− 0.111^♣^	−0.045^♣^	− 0.124^♣^	−0.031^♣^	− 0.127^♣^	−0.117^♣^	0.095^♣^	1	0.185^*^	0.234^**^
	**IL-6 (pg/ml)**	***r***	0.128^♣^	0.227^**^	−0.044^♣^	0.014^♣^	−0.087^♣^	− 0.008^♣^	−0.002^♣^	− 0.025^♣^	0.101^♣^	0.136^♣^	0.059^♣^		1	0.870^**^
	**TNF-α (pg/ml)**	***r***	0.070^♣^	0.170^♣^	0.047^♣^	−0.056^♣^	−0.050^♣^	−0.056^♣^	− 0.022^♣^	−0.003^♣^	0.125^♣^	0.129^♣^	0.087^♣^			1
**G2; Obese group** **(*n*** **= 45)**	**miR-29aR**	***r***	0.219^*^	0.418^**^	−0.586^**^	0.169^♣^	−0.153^♣^	− 0.050^♣^	−0.099^♣^	0.790^**^	−0.091^♣^	− 0.063^♣^	0.718^**^	0.294^**^	0.257^**^	0.443^**^
	**miR-122R**	***r***	0.375^**^	0.639^**^	−0.329^**^	−0.133^♣^	0.231^*^	0.270^**^	0.292^**^	0.387^**^	−0.033^♣^	−0.003^♣^	0.356^**^	0.178^*^	0.201^*^	0.316^**^
	**Sestrin2 (ng/ml)**	***r***	−0.613^**^	−0.341^**^	0.297^**^	0.021^♣^	0.087^♣^	0.129^♣^	0.113^♣^	−0.619^**^	0.121^♣^	0.050^♣^	−0.542^**^	−0.579^**^	− 0.555^**^	−0.674^**^
	**hsCRP (mg/dl)**	***r***	0.632^**^	0.309^**^	−0.328^**^	−0.118^♣^	− 0.048^♣^	0.226^*^	− 0.044^♣^	0.403^**^	− 0.062^♣^	−0.002^♣^	0.364^**^	1	0.475^**^	0.712^**^
	**IL-6 (pg/ml)**	***r***	0.401^**^	0.360^**^	−0.277^**^	− 0.022^♣^	−0.069^♣^	− 0.120^♣^	−0.260^*^	0.468^**^	−0.146^♣^	− 0.042^♣^	0.398^**^		1	0.542^**^
	**TNF-α (pg/ml)**	***r***	0.388^**^	0.399^**^	−0.293^**^	−0.077^♣^	− 0.092^♣^	−0.182^♣^	− 0.118^♣^	0.515^**^	− 0.184^♣^	−0.173^♣^	0.428^**^			1
**G3; Obese with type 2 diabetes** **(*n*** **= 34)**	**miR-29aR**	***r***	0.405^**^	0.474^**^	−0.309^**^	0.635^**^	0.554^**^	0.734^**^	0.574^**^	0.632^**^	0.265^*^	0.466^**^	0.630^**^	0.424^**^	0.464^**^	0.512^**^
	**miR-122R**	***r***	0.631^**^	0.485^**^	−0.346^**^	0.602^**^	0.688^**^	0.607^**^	0.647^**^	0.556^**^	0.591^**^	0.558^**^	0.719^**^	0.488^**^	0.394^**^	0.560^**^
	**Sestrin2 (ng/ml)**	***r***	−0.740^**^	−0.462^**^	0.053^♣^	−0.515^**^	−0.311^**^	− 0.533^**^	−0.797^**^	− 0.603^**^	−0.217^*^	− 0.676^**^	−0.593^**^	− 0.541^**^	−0.600^**^	− 0.558^**^
	**hsCRP (mg/dl)**	***r***	0.757^**^	0.622^**^	−0.291^*^	0.667^**^	0.516^**^	0.625^**^	0.690^**^	0.740^**^	0.238^*^	0.333^**^	0.711^**^	1	0.643^**^	0.773^**^
	**IL-6 (pg/ml)**	***r***	0.691^**^	0.425^**^	−0.234^*^	0.524^**^	0.463^**^	0.510^**^	0.724^**^	0.665^**^	0.338^**^	0.413^**^	0.583^**^		1	0.529^**^
	**TNF-α (pg/ml)**	***r***	0.739^**^	0.581^**^	−0.458^**^	0.656^**^	0.609^**^	0.570^**^	0.711^**^	0.701^**^	0.351^**^	0.673^**^	0.610^**^			1

The values presented in the table are for the correlation coefficient

^♣^ non-significant correlation

^*^correlation is significant at 0.05

^**^correlation is significant at 0.01

WC: waist circumference, BMI: body mass index, HDL-c: high density lipoprotein cholesterol, LDL-c: low density lipoprotein cholesterol, TAG: triacylglycerol, HbA1c: glycosylated hemoglobin, HOMA-IR: Homeostatic model Assessment-Insulin resistance, miR-29a R: microRNA-29a relative expression, miR-122 R: microRNA-122 relative expression, hsCRP: high-sensitivity C-reactive protein, IL-6: interleukin 6, TNF-α: tumor necrosis factor alpha

#### In obese group (G2)

The serum levels of miR-29a and miR-122 correlated positively with WC percentiles, BMI percentiles, HOMA-IR and with serum levels of glucose, hsCRP, IL-6 and TNF-α (Table 3) and negatively with serum HDL-cholesterol (Table 3) and sestrin2 levels (Fig. 2C and D). Also, the serum levels of miR-122 correlated positively with the serum levels of triacylglycerol, total cholesterol and HbA1c% (Table 3). In addition, serum levels of miR-122 and miR-29a correlated positively with each other (Fig. 1B). Moreover, the serum levels of hsCRP, IL-6 and TNF-α correlated positively with WC percentiles, BMI percentiles, HOMA-IR and with serum levels of glucose and negatively with serum levels of HDL-cholesterol (Table 3). On the other hand, serum sestrin2 levels correlated negatively with WC percentiles, BMI percentiles, HOMA-IR and with serum levels of glucose, hsCRP, IL-6 and TNF-α while positively with serum levels of HDL-cholesterol (Table 3).

#### In obese with T2DM group (G3)

The levels of serum miR-29a, miR-122, hsCRP, IL-6 and TNF-α levels correlated positively, while serum sestrin2 levels correlated negatively with WC percentiles and BMI percentiles, and serum levels of LDL-cholesterol, triacylglycerols, HbA1c%, glucose, insulin, c-peptide and with HOMA-IR (Table 3). Also, the serum miR-29a and miR-122 levels correlated positively with each other (Fig. 1B) and with serum hsCRP, IL-6 and TNF-α levels (Table 2). On the other hand, serum sestrin2 levels correlated negatively with the levels of miR-29a and miR-122 (Fig. 2C and D) and with the levels of hsCRP, IL-6 and TNF-α (Table 3).

## Discussion

The significant high levels of the miR-29a, miR-122, IL-6, hsCRP and TNF-α and the low levels of sestrin 2 that were found in obese children with/without T2DM emphasizes that chronic inflammation is a characteristic feature of childhood obesity with/without T2DM [7–10]. Previous studies had reported that the excessive accumulation of fat in the adipocytes leads to the activation of the resident immune cells and initiates a chronic immune-inflammatory process that results in the development of adverse metabolic consequences [15]. Significant increased levels of IL-6, CRP and TNF-α were found by many studies in obese participants compared to the non-obese controls [7, 25, 26]. Also, increased levels of TNF-α and IL-6 along with other proinflammatory cytokines were found in obese children and adolescents with insulin resistance and/or T2DM [25]. The proinflammatory cytokines may induce insulin resistance through unrestrained production of insulin receptor substrate [26].

MicroRNAs play a pivotal role in the modulation and progress of obesity related complications [15]. They have a well-known impact on the oxidant-antioxidant balance, lipid metabolism, and insulin action [27]. Also, they are involved in the development of insulin resistance and the development of T2DM through their effect on the insulin signaling pathway [13, 28]. Moreover, microRNAs act as fine-control signal regulators for inflammatory processes through targeting the expression of the pro-inflammatory and anti-inflammatory genes in the immune cells [29].

In this regard the results of the current study are in accordance with the results of the study conducted by Pandey et al. [2011] who found an increased expression of miR-29a in the db/db mice and this overexpression attenuates the insulin action [30]. Also, they go with the findings of Tang et al. [2017] who reported an ability of miR-29a to enhance the production pro-inflammatory mediators such as IL-6 by targeting the protein kinase B/nuclear factor kappa beta pathway [14].

MiR-29a can downregulate the peroxisome proliferator-activated receptor delta (PPAR δ) in skeletal muscles, which decreases the expression glucose transporter-4 (GLUT-4) and so the insulin dependent glucose entry to the cell [28]. Also, it prevents the repression of phospheneolpyruvare carboxykinase gene by insulin resulting in enhanced gluconeogenesis and marked increase in blood glucose levels [30]. Yang et al. [2014] found that the diet rich in saturated fatty acids resulted in an overexpression of miR-29a which led to a repression in the insulin receptor substrate 1 (IRS-1) and eventually to insulin resistance with decrease in cellular glucose uptake. This repression occurs via the direct binding of the miR-29a to the 3′ untranslated regions of IRS-1 mRNAs [31].

Contrary to the results of the present study, Iacomino and Siani [2017], and Ortega et al. [2014] found a non-significant difference in the miR-29a expression between obese and normal weight individuals [11, 32].

Another microRNA studied in the current work is miR-122. It is an abundant microRNA in the liver and accounts for more than 75% of the total hepatic microRNAs expression [33]. It regulates lipid metabolism (e.g. lipogenesis, cholesterol and VLDL synthesis) through its action on hepatocyte nuclear factor 4α (HNF-4α) [33, 34].

The results of the current study are in agreement with many of the previous studies. Yang et al. [2012] reported that the downregulation in miR-122 induces the protein tyrosine phosphatase 1β which phosphorylates and inactivates HNF-4α resulting in insulin resistance [35]. Jones et al. [2017] observed a positive correlation between miR-122 and insulin resistance and the amount of subcutaneous fat. They concluded that miR-122 might be a good marker for obesity and its related metabolic consequences [36]. Wang et al. [2015] found a 3.07-fold increase in the serum levels of miR-122 of obese patients when compared to healthy non-obese controls and these levels correlated positively with the BMI, serum levels of triacylglycerols, and HOMA-IR. Up on these findings, they suggested a possible role of miR-122 in the pathogenesis of obesity and insulin resistance [37]. Ortega et al. [2010] reported significant high levels of miR-122 in obese participants compared to those with normal weight and these levels decreased markedly after induction of weight loss surgically by gastric bypass operation [38].

Also, the results of the present work are in accordance with the results of Prats-Puig et al. [2013] who found significant high levels of miR-122 in obese children compared to non-obese children. They also observed significant positive correlations between miR-122 levels and BMI, WC, triacylglycerols, LDL and HOMA-IR [39]. In addition, the results work in with the findings of de Candia et al. [2017] who found a significant elevation of miR-122 in patients with impaired glucose tolerance compared to the healthy control group [40]. Moreover, Willeit et al. [2017] revealed significant high levels of miR-122 in adults with T2DM and these levels correlated positively with BMI, WC, triacylglycerols, hsCRP and HOMA-IR [41]. Indeed, miR-122 has been accused in the pathogenesis of many aspects of metabolic syndrome such as T2DM, hypertension, atherosclerosis and even, heart failure [42, 43].

The positive correlations between circulating miR-122 levels and IL-6, hsCRP and TNF-α agree with Song et al. [2020] and Zhao et al. [2020] who concluded that miR-122 controls many immune-inflammatory processes including autophagy, programmed cell death and oxidative stress [43, 44]. Also, go with Wang et al. [2019] who found a significant decrease in the macrophages number and the levels of TNF-α in miR-122 knockout mice [45].

Sesrtins are inducible proteins that protect the cell during stress and inflammatory conditions by enhancing the cellular energy production and stimulation of the genome repair system [18, 46, 47]. Also, they get rid of reactive oxygen species either directly by acting on peroxiredoxins (antioxidant enzymes) or indirectly by regulating the expression of antioxidant genes (e.g. NrF2) [46]. Moreover, sestrins control numerous points in cellular metabolism by their ability to activate the AMP-dependent protein kinase (AMPK) and inactivation of the mammalian target of rapamycin (mTOR) [18, 46, 48]. The inactivation of mTOR markedly decreases the cellular proteins and lipids synthesis and improves the insulin sensitivity by induction of phosphalidyl inositol-3-kinase enzyme (PI3K) [46, 49].

The results of the current work agree with the results of Mohany and Al Rugaie [2020] who found significant lower levels of serum sestrin2 in patients with T2DM than the healthy control group [50]. Also, they agree with Sundararajan et al. [2020] who reported low serum sestrins levels in patients with dyslipidemia and T2DM and these levels correlated negatively with atherogenic factors and the severity of atherogenic index [51]. Moreover, Nourbakhsh et al. [2017] found significant lower levels of sestrin2 in obese than the non-obese participants [19]. Insulin upregulates intracellular sestrin2 content and decreases its degradation through PI3K/mTOR signaling pathway [52]. Lack of normal cellular response to insulin in obese children with T2DM might explain the low sestrin2 levels and their negative correlations with HOMA-IR.

The negative correlations that were found in the present work between serum sestrin 2 levels and the levels of miR-29a and microR-122 reveals the complex nature of the pathogenesis of obesity, T2DM and their accompanied inflammations.

On contrary to the results of the current work, some previous studies reported significant high serum levels of sestrin 2 in obesity with/without T2DM but still showed significant positive correlations with BMI, serum levels of insulin, and HOMA-IR [20]. Also, sestrin 2 in the mice liver was found to be upregulated in obese mice feed on high fat diet [18]. The present study couldn’t explain this inconsistency.

In conclusion, the change in the serum miR-29a, miR-122 and sestrin2 levels in obese children with/without T2DM may suggest a possible role of these biomarkers in the pathogenesis of childhood obesity and their accompanied complications e.g. inflammations and type 2 diabetes mellitus.

### Recommendation

Further studies are required to test the role of miRNAs and sestrin 2 in the pathogenesis of obesity and T2DM and their accompanied inflammation. To prevent these inflammations and other obesity related complications, drugs that antagonize the action miR-29a and miR-122 or upregulate sestrin2 levels should be tested.

## Supplementary Information

**Additional file 1.**

## Data Availability

The datasets used and/or analyzed during the current study are available from the corresponding author on reasonable request. The sequences of miRNA-29a, − 122 and 39 are available @ www.mirbase.org

## Abbreviations

**RNA**: ribonucleic acid
**T2DM**: type 2 diabetes mellitus
**IL-6**: interleukin-6
**hsCRP**: high sensitivity C-reactive protein
**TNF-α**: tumor necrosis factor alpha
**WC**: waist circumference
**BMI**: body mass index
**ELISA**: enzyme-linked immunosorbent assay
**HbA1c%**: glycosylated hemoglobin percentage
**HDLc**: high density lipoproteins cholesterol
**LDLc**: low density lipoproteins cholesterol
**HOMA-IR**: homeostasis model assessment of insulin resistance
**cDNA**: complementary deoxyribonucleic acid
**SPSS**: statistical package for the social sciences
**PPAR**: peroxisome proliferator-activated receptor
**PI3K**: phosphalidyl inositol-3-kinase enzyme
**Glut-4**: glucose transporter-4
**IRS-1**: insulin receptor substrate-1
**HNF-4α**: hepatocyte nuclear factor − 4 alpha
