# Supplementary material for: Investigation of the levels of circulating miR-29a, miR-122, sestrin 2 and inflammatory markers in obese children with/without type 2 diabetes: a case control study
Source: BMC Endocr Disord. 2021 Aug 3;21:152. doi: 10.1186/s12902-021-00829-z (PMC8330040; doi:10.1186/s12902-021-00829-z)
Supplement: Supplementary file 1 — Additional file 1. [file 12902_2021_829_MOESM1_ESM.docx]

**Supplementary 1: Sequences of the analyzed miRNAs**

miRNA-29a: UAGCACCAUUUGAAAUCGGUUA

miRNA-122: UGGAGUGUGACAAUGGUGUUUG

cel-miRNA-39: AGCUGAUUUCGUCUUGGUAAUA

The data are available @ [miRBase](http://www.mirbase.org/) ([WWW.mirbase.org](http://WWW.mirbase.org))
